# Infant temperament contributes to early infant growth: A prospective cohort of African American infants

**DOI:** 10.1186/1479-5868-6-51

**Published:** 2009-08-05

**Authors:** Meghan M Slining, Linda Adair, Barbara Davis Goldman, Judith Borja, Margaret Bentley

**Affiliations:** 1 Department of Nutrition, School of Public Health, University of North Carolina at Chapel Hill, Chapel Hill, NC, USA; 2 Carolina Population Center, University of North Carolina at Chapel Hill, Chapel Hill, NC, USA; 3 Frank Porter Graham Child Development Institute, University of North Carolina at Chapel Hill, Chapel Hill, NC, USA

## Abstract

**Background:**

Prospective studies linking infant temperament, or behavioral style, to infant body composition are lacking. In this longitudinal study (3 to 18 months), we seek to examine the associations between two dimensions of infant temperament (*distress to limitations *and *activity level*) and two anthropometric indicators (weight-for-length z-scores (WLZ) and skin fold (SF) measures) in a population at high risk of overweight.

**Methods:**

Data are from the Infant Care and Risk of Obesity Project, a longitudinal study of North Carolina low income African American mother-infant dyads (n = 206). Two temperament dimensions were assessed using the Infant Behavior Questionnaire-Revised. A high *distress to limitations *score denotes an infant whose mother perceives that s/he often cries or fusses, and a high *activity level *score one who moves his/her limbs and squirms frequently. Cross-sectional analyses were conducted using ordinary least squares regression. Fixed effects longitudinal models were used to estimate anthropometric outcomes as a function of time varying infant temperament.

**Results:**

In longitudinal models, increased *activity levels *were associated with later decreased fatness and WLZ. In contrast, high levels of *distress to limitations *were associated with later increased fatness at all time points and later increased WLZ at 12 months.

**Conclusion:**

Infant temperament dimensions contribute to our understanding of the role of behavior in the development of the risk of overweight in the formative months of life. Identification of modifiable risk factors early in life may help target strategies for establishing healthy lifestyles prior to the onset of overweight.

## Background

Overweight and obesity have increased rapidly in the U.S., especially among African American children. [1,2] Prevention of overweight needs to begin early in life, as childhood overweight often tracks into adulthood [3,4] and is associated with the development of adverse health outcomes both during childhood [5,6] and during later adulthood. [7] Weight gain and body size, even during the early months of life, influence weight status later in childhood, as well as the development of adult cardiovascular and metabolic disease. [8-11]

The determinants of infant somatic growth are not well understood. While parental behavioral and demographic characteristics are often considered salient influences on infant growth patterns (e.g. type and style of infant feeding and socioeconomic resources) [12-15], results are inconsistent. For example, low socioeconomic resources have been shown to contribute either to infant under-nutrition [16,17] or to overweight [2,18]. Furthermore less is known about the contribution of infant characteristics to parental feeding and care behaviors that may affect infant physical growth.

Conceptually there are at least three pathways (mechanisms) by which aspects of infant behavior could influence growth: (a) an infant's difficult or distressed behavior may contribute to the initiation of caretaking activities such as feeding that increase energy intake [19]; (b) an infant's difficult or distressed behavior may be expressed through crying and fussing which expend energy [20]; or (c) the amount and vigor of an infant's motor activity behavior may increase energy expenditure. Given that infant-initiated behavior may affect both sides of the energy balance equation, the role of infant behavioral style, or temperament, merits investigation.

Infant temperament refers to biologically rooted differences in behavior believed to be present early in life and relatively stable across time and context. [21] Temperament is composed of a number of traits or dimensions including fear, positive and negative affect, sadness, distress to limitations (crying and fussing) and activity level. Two of these temperament dimensions may be of particular importance for understanding variability in infant somatic growth through the pathways discussed above: *distress to limitations *(pathways a and b) and *activity level *(pathway c).

Most evidence suggests that "difficult" temperament, a broader concept which includes considerable to excessive fussing and crying, or *distress to limitations (DTL)*, is related to increased weight gain and body size as proposed in pathway (a) above. [19,22,23] Two studies have related "difficult" temperament to increased weight gain in infancy. The first determined that difficult temperament, measured at 6 months of age, was slightly positively associated with rapid weight gain during the first 6 months of life [22] while the second showed that 12-month-old infants with the greatest weight gain had significantly more difficult temperament ratings at 6-months of age. [19] A third study found that *DTL *was related to fast gain in the first eight weeks of life. [23] While Wells et al. [20] did not find a relationship between *DTL *and infant fatness, their group found that higher *DTL *was associated with significantly higher levels of energy expenditure as proposed in pathway (b) above.

In contrast, the extant literature on the relationship between *activity level *and body size and composition is equivocal. Two studies showed an association between activity level and size, although in opposite directions. One found that heavy infants spent more time moving their arms and legs vigorously than did the light infants [24] while another found that leg activity was inversely associated with weight among female infants as proposed in pathway (c) above. [25] Conversely, two studies showed no association between activity level and size or body composition. One found that low activity levels at 6 months of age did not lead to higher body fatness at 12 months of age [26] while a second found that infants with the most decelerated growth from 6 to 12 months of age were not more active than those gaining the most. [19]

Previous studies on the relationship between temperament and body size have primarily focused on middle-class, white populations and have largely utilized cross-sectional study designs. Longitudinal research is needed to improve our understanding of the early development of infant behavior patterns and how these patterns contribute to the development of infant size and body composition in infancy.

The objective of the current study was to examine the longitudinal associations from 3 to 18 months of 2 dimensions of infant temperament, *DTL *and *activity level*, with body size and fatness among low income African-American infants, a group at high risk for the development of obesity.

## Methods

### Study Design and Participants

We used data from the Infant Care, Feeding and Risk of Obesity Study (hereafter called Infant Care), a prospective cohort study that assessed mother/infant dyads from 3 to 18 months postpartum. The Infant Care study was primarily designed to examine, in the home environment, how parenting and infant feeding styles relate to infant diet, and the risk of infant overweight. First time African-American mothers aged 18–35 years and their 3-month-old infants were recruited through Women Infants and Children (WIC) clinics in North Carolina and were assessed during in-home visits when infants were approximately 3, 6, 9, 12, and 18 months of age. A total of 217 mothers and infants were recruited at baseline (3 months). Data collection was conducted from 2003–2007. For inclusion, mothers had to be willing to participate in home visits and assessments, and had healthy infants who had completed >35 weeks' gestation. Infants with Down syndrome, epilepsy, cleft lip or palate, cerebral palsy, failure to thrive, mental retardation, severe food allergies or any condition that might affect appetite, feeding or growth were excluded from the study.

The analysis sample includes 206 infants (96 males, 110 females) with data on weight, length, dietary intake and temperament variables of interest. The protocol was approved by the School of Public Health Institutional Review Board at the University of North Carolina at Chapel Hill.

### Study Measurements and Variables

Anthropometrics. Study personnel were trained in standard anthropometric techniques. Anthropometrics were measured at infant ages 3, 6, 9, 12 and 18 months. Weight was measured on a digital scale (Tanita BD-585 Digital Baby Scale) to the nearest 10 g. Recumbent length was measured by a 2-person team, using a portable rigid length board (O'Leary Length Board) to the nearest 0.1 cm. Infant skin fold thicknesses were measured using Harpenden calipers. All anthropometric measurements were done in triplicate and the mean was used in analyses. Weight-for-length z-scores (WLZ) were calculated using the CDC/NCHS 2000 growth reference [27]. Change in WLZ was calculated as a simple difference between subsequent WLZ measures. The main outcome variables of interest were WLZ and the sum of three skin fold (SF) measures (subscapular, abdominal and triceps). Birth weight and gestational age were obtained from maternal report.

Infant Temperament. Temperament refers to constitutionally-based individual differences in reactivity and self-regulation. [28] The continuity of temperamental characteristics over time and in different contexts is among the hallmarks of temperament theory. [29] Temperament was measured by maternal reports of the frequency of the occurrence of temperamentally relevant infant behaviors along a 7-point Likert-type scale across a number of temperamental dimensions.

At infant age 3, 6 and 9 months we examined 2 subscales of the Infant Behavior Questionnaire-Revised (IBQ-R) [30], *distress to limitations (DTL) *and *activity level*. The *DTL *subscale consists of 16 items, which characterize an infant's fussiness, crying and exhibition of distress in different situations. A high *DTL *score denotes an infant that often cries or fusses. Scores range from 1.6 to 5.8 in our sample (with a possible range of 1–7). The *activity level *subscale consists of 15 items, which capture movement of the infant's arms and legs, squirming and locomotor activity. A high activity score on the IBQ-R reflects an infant who squirms and moves his/her limbs often. Scores range from 1.9 to 6.3 in our sample (with a possible range of 1–7). The complete IBQ-R consists of 14 subscales. The validity of the IBQ-R scales has been reported elsewhere [31,32].

At infant age 12 and 18 months we used the toddler version of the IBQ-R, the Early Childhood Behavior Questionnaire (ECBQ). [33] The ECBQ does not include *DTL*. The *activity level *toddler subscale consists of 12 items, which capture level of gross motor activity, including rate and extent of locomotion. A high activity level score reflects a toddler who splashes when bathing, moves quickly from one place to another, seems full of energy, and is generally in motion rather than still. Scores range from 3.4 to 7.0 in our sample. The complete ECBQ consists of 18 subscales.

Given that the IBQ-R and ECBQ were developed using a predominantly Caucasian, middle class sample, [30] concern may be raised about the validity of this measure in our sample population. Four recent studies [34-37] used the IBQ-R with samples which included significant numbers of African-American families, single mothers, and low income families. While a confirmatory factor analysis has been conducted for US and Russian infants [38], to our knowledge a similar confirmatory factor analysis has not been conducted to examine whether the temperament subscales hold together in the same way for an African American sample. Cronbach's alpha coefficients were calculated for temperament subscales at each time point to assess internal consistency of the IBQ-R and ECBQ in our sample (range 0.69–0.78). All eight alphas calculated (2 subscales, each at 4 time points) exceeded 0.60, the value considered the threshold for adequate internal consistency by DeVillis. [39]

Dietary intake. Three 24-hour dietary recalls were completed at each time point using the Nutrition Data System (version 2005 Nutrition Coordinating Center, University of Minnesota, Minneapolis). The first 24-hour recall at each time point (3, 6, 9, 12 and 18 months) was completed during the home visit and was followed by two random nonconsecutive 24-hour recalls via telephone within a 2-week interval following the home visit. Food models and pictures were used during the assessment to aid in the estimation of portion sizes. Approximately 40 infant foods, including commercially prepared baby foods and cereals, were added to the digital food library of the Clinical Nutrition Research Unit at the University of North Carolina at Chapel Hill. Information on breast feeding was collected during in-home interviews. In all models, dietary intake was represented by the inclusion of three variables concurrently: a binary main effect breast feeding variable at the time of each visit (breast fed, not breast fed), a continuous main effect total energy intake variable representing energy intake from non-breast milk substances and a total energy intake by breast feeding interaction term. The latter is needed to account for the fact that energy intake was not measured for breast milk.

Covariates. We considered several covariates including: child sex, birth weight, and gestational age; maternal age, education, and BMI. We did not include income, since this information was not provided by more than half of the sample women. The current literature was the basis for selecting and evaluating these variables. [12,13,40-43]

### Statistical Analysis

The analysis was conducted using STATA (version 10.0, College Station, TX) [44]. Descriptive statistics were calculated for anthropometrics, temperament measures and select sociodemographic factors. We used t- and chi-square statistics to test for differences between males and females for anthropometrics and temperament characteristics. Preliminary analyses suggested a non-linear relationship between the *DTL *temperament subscale and anthropometry. At each time point, we examined the shape of the relationships using locally weighted regression, or loess smoothing graphs [45]. Categorical variables (low, medium and high temperament subscale scores) were then created based on cut points where the slope of the relationship between *DTL *and anthropometry changed. Pearson correlations were computed to examine continuity of temperament measures between time points for each subscale separately. Effect measure modification by sex was examined by testing interaction terms using likelihood ratio tests, with α = 0.15. Mediation by infant diet was examined by comparing crude and diet adjusted cross-sectional models. Evidence for mediation was provided if the unique contribution of infant temperament to the anthropometric outcome was reduced and accounted for by diet, the presumed mediating variable [46]. Tests for confounding were conducted using an *a priori *change in estimate criterion (change in main effect coefficient of >10%).

Cross-sectional analyses were conducted at each time point using ordinary least squares regression models. Ordinary least squares longitudinal regression models with fixed effects methods were used to estimate anthropometric outcomes as a function of time varying diet and lagged infant temperament [47]. Lagged infant temperament variables were used so that inferences about potentially causal sequences could be drawn (i.e. to study how temperament predicts subsequent SF or WLZ). Preliminary cross-sectional analyses indicated that effects were not consistent over time and therefore effect measure modification by visit was examined by testing interaction terms using likelihood ratio tests, with α = 0.15. All models controlled for infant birthweight, gestational age, total energy intake, breastfeeding status and the interaction of breastfeeding with energy intake. To facilitate interpretation of results, WLZ values were predicted for low, medium and high temperament scores at each time point, holding all other variables at their means.

## Results

Table 1 presents characteristics of the mothers and infants in our sample. At all time points, mean WLZ scores were positive, indicating that on average relative weight was higher than the 50^th ^percentile of the CDC/NCHS 2000 growth reference, with approximately 70% of the sample having positive WLZ at every time point. Mean WLZ and the mean sum of skinfolds (SF) decreased slightly from 3–18 months of age. In contrast, means of the mother's scores reflecting her perception of the infant's *DTL *and *activity level *increased slightly from 3–9 and 3–18 months of age, respectively. There were no statistically significant sex differences in any of the anthropometric or temperament measures. Compared to previous studies on temperament and weight status, [20,22,23] the mothers in our sample had higher BMI, fewer years of education and lower rates of breastfeeding (Table 1).

**Table 1:** Descriptive characteristics of mothers and infants from 3–18 months of infant age

	Birth	3 months	6 months	9 months	12 months	18 months
		
**Infants**						
Total n		206	157	153	132	109
Female		110 (53.4%)^a^	83 (52.9%)	80 (52.3%)	73 (55.3%)	59 (54%)
Gestational age	39.48 ± 1.47^b^					
						
***Anthropometrics***						
Birthweight	3.23 ± 0.48					
Weight for length Z-score		0.56 ± 1.0	0.55 ± 1.1	0.54 ± 1.12	0.44 ± 1.08	0.34 ± 1.04
Sum of skinfolds^c^		25.34 ± 4.69	25.53 ± 5.91	24.80 ± 6.33	23.09 ± 5.38	22.88 ± 4.19
						
***Temperament dimensions***						
Distress to limitations		3.46 ± 0.74	3.66 ± 0.80	4.02 ± 0.77	n/a	n/a
Activity level		4.11 ± 0.82	4.71 ± 0.77	4.85 ± 0.73	5.50 ± 0.71	5.35 ± 0.77
						
***Infant Diet***						
Daily calories, non-breastfed infants						
Infant received any breast milk		44 (21.4%)	22 (14.0%)	15 (9.8%)	8 (6.1%)	3 (2.8%)
Infant exclusively breast-fed		13 (6.3%)	0	0	0	0
						
**Mothers**						
						
*Anthropometrics*						
BMI<25		63 (29%)				
BMI 25–29.9		58 (26.7%)				
BMI = >30		96 (44.2%)				
						
*Sociodemographics*						
Maternal education		58 (27.1%)				
Less than high school		65 (30.4%)				
High school graduate or GED		70 (32.7%)				
Some college		21 (9.8%)				

^a ^Frequency (%)

^b^Mean ± standard deviation

^c ^Sum of 3 skinfolds: subscapular, abdominal and tricep

Temperament subscales were moderately stable over time. Pearson correlations between the individual subscales of infant temperament measured at multiple time points during infancy ranged from 0.25 to 0.60 and were all significant at *p *< .01.

The results of the cross-sectional analyses are presented in Tables 2 and 3. There was no strong evidence that diet mediates the relationship between temperament and WLZ or SF; the temperament coefficients were not attenuated by inclusion of dietary variables. As diet was not found to be a mediator it was included as a covariate in all models. *Activity level *was a significant predictor of SF at 3 months only (Table 2). Infants whose mothers perceived them to be more active had lower SF (β = -0.93, p = 0.02). *Activity level *was not a significant predictor of WLZ when measured concurrently at any time point. Preliminary analysis of the crude relationship between *DTL *and anthropometry suggested that it was nonlinear. *DTL *was not a significant predictor of either WLZ or SF (Table 3) at any point in the cross-sectional analyses.

**Table 2:** Cross-sectional Results: Activity Temperament Dimension and Infant Anthropometry

	***3 month visit***		***6 month visit***		***9 month visit***		***12 month visit***		***18 month visit***	
	*n*	β (95% CI)^a^	*n*	β (95% CI)	*n*	β (95% CI)	*n*	β (95% CI)	*n*	β (95% CI)
***Weight-for-length Z-scores***										
Activity Level^b^	206	-0.11 (-28, 0.05)	157	0.07 (-0.17, 0.30)	153	0.14 (-0.11, 0.39)	132	-0.03 (-0.30, 0.24)	109	0.13 (-0.13, 0.39)
										
***Sum of Skinfold Measures***										
Activity Level	206	-0.93 * (-1.70, -0.16)	157	-0.43 (-1.67, 0.82)	153	0.09 (-1.35, 1.53)	132	-0.84 (-2.45, 0.76)	109	1.01 (-0.09, 2.11)

Adjusted for age at measurement, total calorie intake, breastfeeding status, birthweight, gestational age and sex.

^a ^Beta coefficient and 95% Confidence Interval

^b^Activity level subscale from the Infant Behavior Questionnaire-Revised

* *P *< 0.05

**Table 3:** Cross-sectional Results: Distress to limitations Temperament Subscale and Infant Anthropometry

	***3 month visit***		***6 month visit***		***9 month visit***	
	*n*	β (95% CI)^a^	*n*	β (95% CI)	*n*	β (95% CI)
***Weight-for-Length Z-scores***						
Low DTL^b^	48	-0.19 (-0.53, 0.16)	33	0.26 (-0.25, 0.78)	33	0.28 (-0.22, 0.79)
Mid DTL (referent)	109	0	84	0	82	0
High DTL	49	0.15 (-0.20, 0.49)	39	0.01 (-0.43, 0.45)	38	0.24 (-0.20, 0.67)
***Sum of 3 Skinfold Measures***						
Low DTL	48	0.62 (-0.99, 2.23)	33	0.66 (-2.15, 3.47)	33	2.80 (-0.08, 5.68)
Mid DTL (referent)	109	0	84	0	82	0
High DTL	49	1.32 (-0.28, 2.92)	39	0.12 (-2.26, 2.50)	38	1.78 (-0.70, 4.25)

Adjusted for age at measurement, total calorie intake, breastfeeding status, birthweight, gestational age and sex.

^a ^Beta coefficient and 95% Confidence Interval

^b^Distress to Limitations (DTL) subscale from the Infant Behavior Questionnaire-Revised

**Figure 1:**
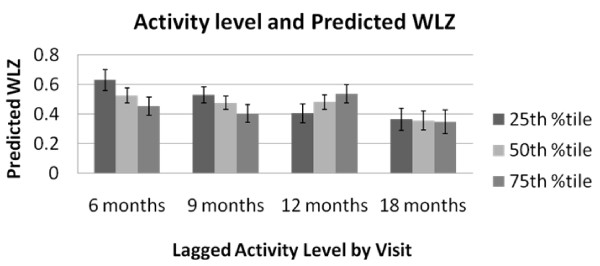
Predicted Weight-for-length Z-score by lagged 25^th^, 50^th^, and 75^th ^percentiles of Activity* Level and infant age. * Activity level subscale from the Infant Behavior Questionnaire-Revised.

The results of the fixed effects longitudinal analyses are presented in Tables 4 and 5. The relationship between lagged temperament and WLZ was found to change over time as indicated by the significant visit × temperament interaction terms. There was no evidence of a significant interaction between lagged temperament and visit in SF models, indicating that the associations did not differ by age of the child. *Activity level *was a significant predictor of subsequent fatness and relative weight (Table 4). Higher *activity level *was associated with lower subsequent SF–a one unit increase in maternal perception of infant activity level was associated with a 0.61 unit decrease in SF (β = -0.61, p < 0.05). Similarly, higher *activity level *was associated with lower subsequent WLZ at nearly all time points with the exception of higher *activity level *at 9 months of age being associated with higher subsequent WLZ at 12 months (Figure 1).

**Table 4:** Regression results examining overall (fixed) effects of Lagged Activity on Anthropometry

***OUTCOME = Weight-for-length Z-scores***	
Variable	β (95% CI)^b^
	
Lagged Activity^a^	-0.16 (-0.29, -0.02)*
Total calories per day	-0.01 (-0.01, 0.01)
Breastfed (yes/no)	-0.27 (-0.70, 0.17)
Breastfed status by total calories interaction term	0.01 (-0.01, 0.01)
9 month visit	-0.07 (-0.85, 0.71)
12 month visit	-1.36 (-2.24, -0.48)*
18 month visit	-0.71 (-1.77, 0.34)
Lagged Activity by 9 month visit interaction term	0.02 (-0.15, 0.19)
Lagged Activity by 12 month visit interaction term	0.29 (0.10, 0.48)*
Lagged Activity by 18 month visit interaction term	0.14 (-0.07, 0.35)
Intercept	1.32 (0.71, 1.94)*
	
***OUTCOME = Sum of Skinfold Measures***	

Variable	β (95% CI)^b^
	
Lagged Activity	-0.61 (-1.19, -0.03)*
Total calories per day	-0.01 (-0.01, 0.01)
Breastfed (yes/no)	2.13 (-0.53, 4.79)
Breastfed status by total calories interaction term	-0.01 (-0.01, 0.01)
9 month visit	-0.51 (-1.35, 0.33)
12 month visit	-1.21 (-2.21, -0.21)*
18 month visit	-1.57 (-2.92, -0.22)*
Intercept	28.21 (25.57, 30.86)*

^a ^Lagged Activity level subscale from the Infant Behavior Questionnaire-Revised

^b ^Beta coefficient and 95% Confidence Interval

* P < 0.05

**Table 5:** Regression results examining overall (fixed) effects of Lagged Distress to Limitations on Anthropometry

***OUTCOME = Weight-for-length Z-scores***	
Variable	β (95% CI)^b^
	
Lagged Low Distress to Limitations^a^	-0.06 (-0.34, 0.22)
Lagged High Distress to Limitations^a^	-0.04 (-0.29, 0.21)
Total calories per day	-0.01 (-0.01, -0.01)*
Breastfed (yes/no)	-0.31 (-0.84, 0.21)
Breastfed status by total calories interaction term	0.01 (-0.01, 0.01)
9 month visit	-0.04 (-0.21, 0.12)
12 month visit	-0.19 (-0.40, 0.01)
Lagged Low Distress to Limitations by 9 month visit interaction term	-0.05 (-0.43, 0.33)
Lagged Low Distress to Limitations by 12 month visit interaction term	0.15 (-0.25, 0.55)
Lagged High Distress to Limitations by 9 month visit interaction term	0.01 (-0.33, 0.34)
Lagged High Distress to Limitations by 12 month visit interaction term	0.45 (0.09, 0.80)*
Intercept	0.82 (0.56, 1.08)*
	
***OUTCOME = Sum of Skinfold Measures***	

Variable	β (95% CI) b
	
Lagged Low Distress to Limitations	0.67 (-0.54, 1.89)
Lagged High Distress to Limitations	1.30 (0.29, 2.31)*
Total calories per day	0.01 (-0.01, 0.01)
Breastfed (yes/no)	2.40 (-0.78, 5.59)
Breastfed status by total calories interaction term	-0.01 (-0.01, 0.01)
Intercept	24.86 (23.41, 26.32)*

^a^Lagged Distress to Limitations subscale from the Infant Behavior Questionnaire-Revised

^b^Beta coefficient and 95% Confidence Interval

* P < 0.05

**Figure 2:**
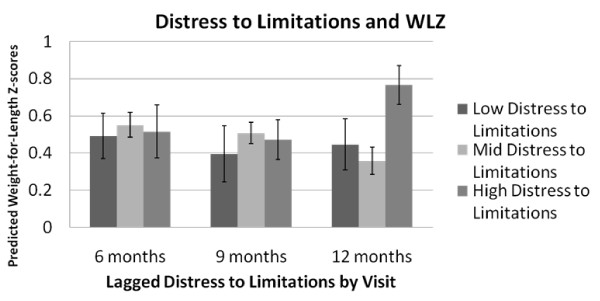
Predicted Weight-for-length Z-score by lagged Distress to Limitations* level and infant age. * Distress to limitations subscale from the Infant Behavior Questionnaire-Revised.

*Distress to limitations *was a significant predictor of subsequent fatness and relative weight (Table 5). As compared to infants with moderate *DTL*, infants with high levels of *DTL *had higher subsequent SF (β = 1.30, p = 0.01) at all subsequent visits. However, high, as compared to moderate levels of *DTL *were only associated with subsequent higher WLZ at 12 months (Figure 2).

## Discussion

### Summary of main findings

Few studies have examined the longitudinal associations between infant temperament and body composition in infancy. This study was conducted to examine the relationship between two temperament subscales and two anthropometric indicators at 3, 6, 9, 12 and 18 months of infant age in a low-income, African American sample at high risk of overweight. Results from cross-sectional and fixed effects longitudinal models differed remarkably. In longitudinal models, increased *activity levels *were associated with decreased fatness at all time points and with decreased WLZ at nearly all time points, while in cross-sectional models *activity level *was a significant predictor of fatness at only 3 months. High levels of *DTL *were associated with increased fatness at all time points and increased WLZ at 12 months in the longitudinal models while in cross-sectional models high levels of *DTL *were not associated with anthropometry at any time point.

### Activity Level

Higher *activity level *was associated with later decreased fatness. A similar relationship was observed in which higher *activity level *was associated with later decreased WLZ except at infant age 12 months where the direction of the relationship changed. Similar to our results, Worobey found that leg activity was inversely associated with weight among female infants. [25] In contrast, an early study by Pollitt and colleagues found that heavy Indonesian infants spent more time moving their arms and legs vigorously than did the light infants. [24] Two additional studies showed no association between activity level and subsequent body fatness or weight gain. [19,26]

### Distress to limitations

We found an association between high levels of *DTL *and increased anthropometry, specifically that high *DTL *was associated with subsequent increased SF values, more in line with hypotheses that would predict increased intake due to efforts to calm fussy infants via feeding. Similarly, high, as compared to moderate levels of *DTL *were also associated with subsequent increased WLZ, although only at 12 months of infant age. In a cross-sectional study of 50 three-month-old infants Wells et al. found that higher *DTL *was associated with significantly higher levels of energy expenditure as measured with doubly-labeled water [20], but that *DTL *was not related to infant fatness. [48] In contrast, other studies before and after Wells et al. have related difficult temperament (which includes fussiness) to increased weight gain in infancy. [19,22,23] One large recent study determined that difficult temperament at 6 months of age was just slightly positively associated with previous rapid weight gain (birth to 6 months) [22] while a much smaller, earlier study showed an association between increased weight for length gain between 6 and 12 months and difficult temperament in the same time period. [19] A third study found that the temperament dimension *DTL *measured at 8 weeks of infant age was related to previous fast weight gain (birth to 8 weeks). [23] In our sample we did not find any relationship between *DTL *and change in weight-for-length z-score (results not shown).

### Interpretation and Implications

Two strengths of the current study are the longitudinal nature of the data and the use of lagged temperament variables. Results from cross-sectional and longitudinal models differed considerably. Unobserved mother and infant characteristics may have influenced both temperament and anthropometry. Over time this unobserved heterogeneity could bias estimates from cross-sectional models. The strength of the fixed effects longitudinal model is that it can eliminate such bias.

Furthermore, the use of lagged temperament variables allows the examination of directionality and temporality in the longitudinal models. One might hypothesize that infant physical growth influences parental perceptions of their infants' temperament. To model the effects of infant temperament on infant anthropometry, we specified our models with lagged infant temperament variables, that is, temperament measured at the visit *prior to *the measurement of infant anthropometry.

Inconsistencies between our results and earlier studies may be attributed to the longitudinal nature of our data as well as the use of lagged exposure variables which capture directionality. In addition, the same infant behavior, such as high levels of crying and fussiness, may lead to *both *increased energy expenditure on the part of the infant, and increased intake via additional feedings in attempts to soothe on the part of the parent of that same infant. While we did not find strong evidence that diet mediates the relationship between temperament and WLZ or SF in our sample it has been suggested that persistent, frequent or intense fussy infant behavior which is perceived as difficult may prompt at least some caregivers to use feeding as a strategy to calm distressed infants. [19] Depending on whether the increased intake balances out or exceeds the increased expenditure, different effects on body size and composition at different moments in time in the same infant, and across infants may be observed.

Importantly, our results suggest that infant activity may have differential effects on relative fatness and body size by developmental status. As expected, infants who were perceived by their mothers to have higher activity levels had considerably lower body fat at all subsequent time points. The relationship between activity level and subsequent WLZ scores, however, changed direction at 12 months of infant age. Taken together, these results suggest that infant body composition changes as infant motor development and mobility changes. Infants with higher activity levels are less fat but are more likely gaining muscle weight (as suggested by increased WLZ) when they become more mobile at 12 months of age.

### Limitations

While this dataset offers a unique opportunity to provide information about low-income African American infants, a population at high risk of overweight, the results from this study may not apply to all U.S. infants. Furthermore, comparison of our results with previous studies may be limited by the dissimilarities of the study populations.

All temperament measures are based on maternal reports: Mothers' perception of infant temperament and behavior may differ by sex, education, or mothers' BMI in other samples, even though we did not find that here.

## Conclusion

In a prospective cohort study of low income African-American infants, a group at high risk for the development of obesity, maternal perceptions of infant temperament, or behavioral style significantly contributed to subsequent infant fatness and relative weight, as measured by sum of skin folds and WLZ. In general increased *activity levels *were associated with later decreased infant skin fold measures and WLZ while increased DTL was associated with later increased infant skin folds. Infant temperament contributes to our understanding of the behavioral contributions to the early development of child fatness and the risk of overweight in the formative months of life. Further research is needed to identify modifiable mediating variables. Identification of such risk factors early in life may help target strategies for establishing healthy lifestyles prior to the onset of overweight.

## Abbreviations

DTL: distress to limitations; WLZ: weight-for-length z-score; SF: skin fold.

## Competing interests

The authors declare that they have no competing interests.

## Authors' contributions

MS conducted all analyses and drafted the manuscript. LA participated in the design of the study and participated in statistical analyses. BDG participated in the design of the study, interpretation of temperament variables and helped to draft the manuscript. JB participated in the design of the study. PB conceived of the study, and participated in its design and coordination and helped to draft the manuscript. All authors read and approved the final manuscript.
